# ALE: automated label extraction from GEO metadata

**DOI:** 10.1186/s12859-017-1888-1

**Published:** 2017-12-28

**Authors:** Cory B. Giles, Chase A. Brown, Michael Ripperger, Zane Dennis, Xiavan Roopnarinesingh, Hunter Porter, Aleksandra Perz, Jonathan D. Wren

**Affiliations:** 1 Arthritis & Clinical Immunology Program, Oklahoma Medical Research Foundation, 825 N.E. 13th Street, Oklahoma City, OK 73104 USA; 2 Department of Biochemistry and Molecular Biology, University of Oklahoma Health Sciences Center, Oklahoma City, OK USA; 3 Vanderbilt University, 2201 West End Ave, Nashville, TN USA; 4 Department of Computer Science, Baylor University, Hankamer Academic Building, 105 Baylor Ave, Waco, TX 76706 USA

**Keywords:** Gene expression omnibus, Gene expression, Text mining, Meta-analysis

## Abstract

**Background:**

NCBI’s Gene Expression Omnibus (GEO) is a rich community resource containing millions of gene expression experiments from human, mouse, rat, and other model organisms. However, information about each experiment (metadata) is in the format of an open-ended, non-standardized textual description provided by the depositor. Thus, classification of experiments for meta-analysis by factors such as gender, age of the sample donor, and tissue of origin is not feasible without assigning labels to the experiments. Automated approaches are preferable for this, primarily because of the size and volume of the data to be processed, but also because it ensures standardization and consistency. While some of these labels can be extracted directly from the textual metadata, many of the data available do not contain explicit text informing the researcher about the age and gender of the subjects with the study. To bridge this gap, machine-learning methods can be trained to use the gene expression patterns associated with the text-derived labels to refine label-prediction confidence.

**Results:**

Our analysis shows only 26% of metadata text contains information about gender and 21% about age. In order to ameliorate the lack of available labels for these data sets, we first extract labels from the textual metadata for each GEO RNA dataset and evaluate the performance against a gold standard of manually curated labels. We then use machine-learning methods to predict labels, based upon gene expression of the samples and compare this to the text-based method.

**Conclusion:**

Here we present an automated method to extract labels for age, gender, and tissue from textual metadata and GEO data using both a heuristic approach as well as machine learning. We show the two methods together improve accuracy of label assignment to GEO samples.

## Background

**Fig. 1:**
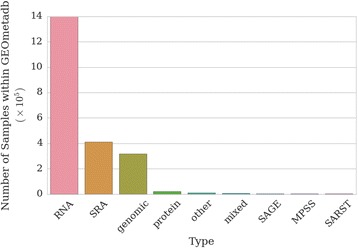
Distribution of molecule types in GEO. GEO consists of a variety of assay types, most predominantly RNA expression quantification by array. Note the Y-axis is in units of 10^5^. Data retrieved and analyzed using database and software from [9]

**Fig. 2:**
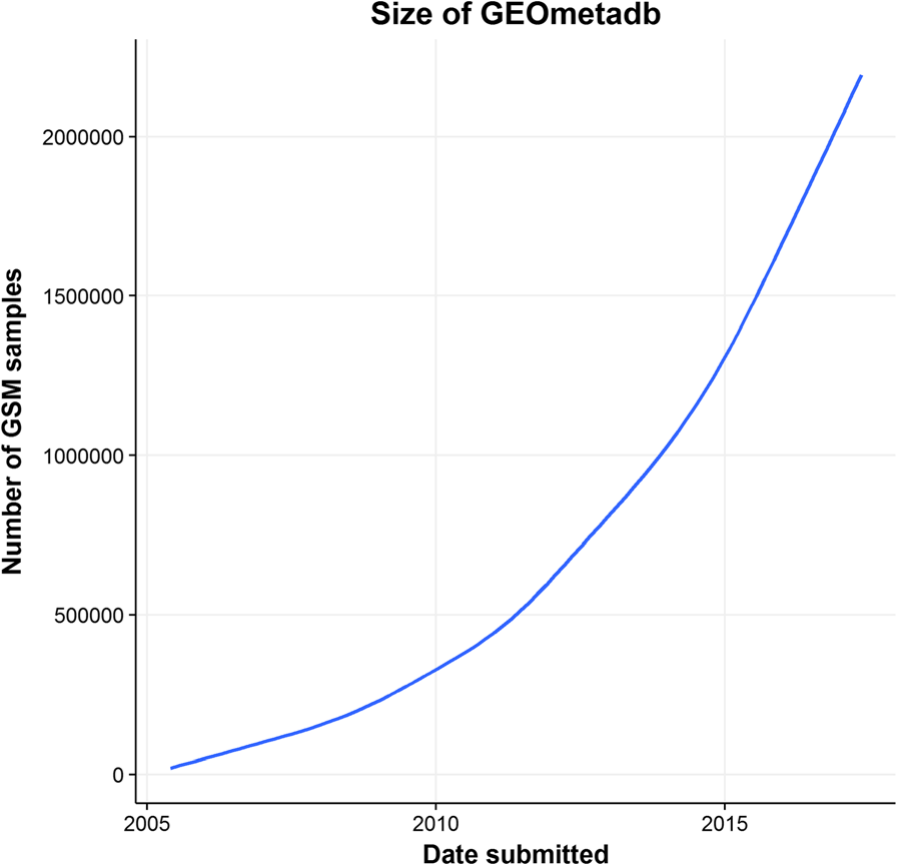
Size increase of GEOmetadb over time. Number of Data Samples from GEOmetadb. Data retrieved and analyzed using database and software from a GEOmetadb package in R [9]

The NCBI Gene Expression Omnibus [1] is a large, public repository of high-throughput genomic datasets that archives experimental data from investigators around the world investigating a variety of species, diseases, and experimental conditions. It has served as a primary resource for investigators to query past experiments to answer specific questions, as well as a data source for large-scale meta-analyses [2]. Although GEO, as indicated by its name, was originally created to archive gene expression microarray experiments, it has grown in scope to include data from methylation arrays and high-throughput sequencing experiments, among other data types (Figs. 1 and 2). As GEO continues to grow rapidly in size, it remains a relevant and important source of data even as the biomedical research community shifts from array-based to sequencing-based approaches.

For each archived experimental sample, GEO provides both the data itself, consisting of a vector of counts or probe intensities, as well as the metadata associated with the sample. The metadata usually contains valuable information regarding the nature of the sample, for example the species of origin, array or sequencing platform, as well as the age, gender, tissue of origin, and experimental perturbation(s), mutations, or disease state applied to the sample. Unfortunately, most of this metadata is not provided in a standardized format directly amenable to larger-scale analyses. Rather, it is provided as free-text descriptions which are optionally provided by the investigator. Therefore, these textual fields have a potential to be missing, misspelled, or described in a variety of ways with various synonyms and identifiers. For example, the binary variable representing “gender” can be described in a variety of ways, e.g., “M”, “male”, “1” (in 0-1 coding), and so on. For other fields which may contain a larger variety of values, such as tissue type, the heterogeneity is even larger. This heterogeneity in data documentation in the GEO metadata causes difficulty or completely disables the ability to query the GEO database effectively for large scale comparisons of this large amount of biological data.

Furthermore, in compiling our gold-standard for annotation of GEO records, we found 86% of metadata descriptors contained tissue information provided by the investigator, while only 26 and 21% contained gender and age information respectively. This suggests that a machine-learning approach to predicting age and gender labels is needed in addition to text-based classification, because there is otherwise no way of obtaining this data apart from time-consuming measures such as contacting the original investigator on a case-by-case basis. Therefore, we present a system which enables researchers to more easily obtain labels and compare datasets within GEO via similar groupings such as age, tissue, and sex.

### Similar work

The need for automated metadata structuring and error correcting in large biological databases has been acknowledged within the field, and there have been attempts to ameliorate these problems with several tools in the past few years. Various methods have been developed to infer labels from GEO for downstream meta-analysis or other large-scale uses of GEO data whereby the sheer volume of samples makes it infeasible to manually curate labels for all samples. Crowdsourcing is one means of doing this cheaply, but will require continued effort as new data comes out [3].

Methods have also been developed to not only extract labels from text, but to *infer* the labels from the gene expression data itself. Lee et al. developed URSA (Unveiling RNA Sample Annotation) as an automated method, which utilized one-vs-all or one-vs-rest (OVR) support vector machines (SVMs) on gene expression data in order to infer labels from the gene expression data [4]. They then mapped the SVMs to the directed acyclic graph (DAG) of the BRENDA Tissue Ontology and assigned the probability of being associated with a certain class by selecting the highest Bayesian conditional probability.

Buckberry et al. developed a method to infer sex from gene expression data by clustering the expression data, and inferring the labels from the expression of Y chromosomes. Unfortunately, this assumes that the data consists of samples from both sexes, and that the Y chromosome expression is one of the main data features for which the data will cluster [5]. Other works have focused on finding semantic similarity between ontologies and metadata in ChIP-seq data from GEO, or have broadened their impact to several different database sources including PubMed, ArrayExpress, GEO, and others [6].

In addition to label extraction from GEO, a recent study has provided a tool for label extraction from the Sequence Read Archive (SRA) metadata as well [7]. The database yielded from this work (MetaSRA) was created using a slightly different set of algorithms in order to achieve a goal similar to the GEO metadata projects. First, they structure the database schema similarly to the schema in the ENCODE project [8]. The MetaSRA system is constructed by mapping terms to ontologies, which is comparable to the methods used within the work we present here; however, the MetaSRA system uses filtering mechanisms for the mapped ontologies which delineate term mentions vs. term mappings.

## Methods

**Fig. 3:**
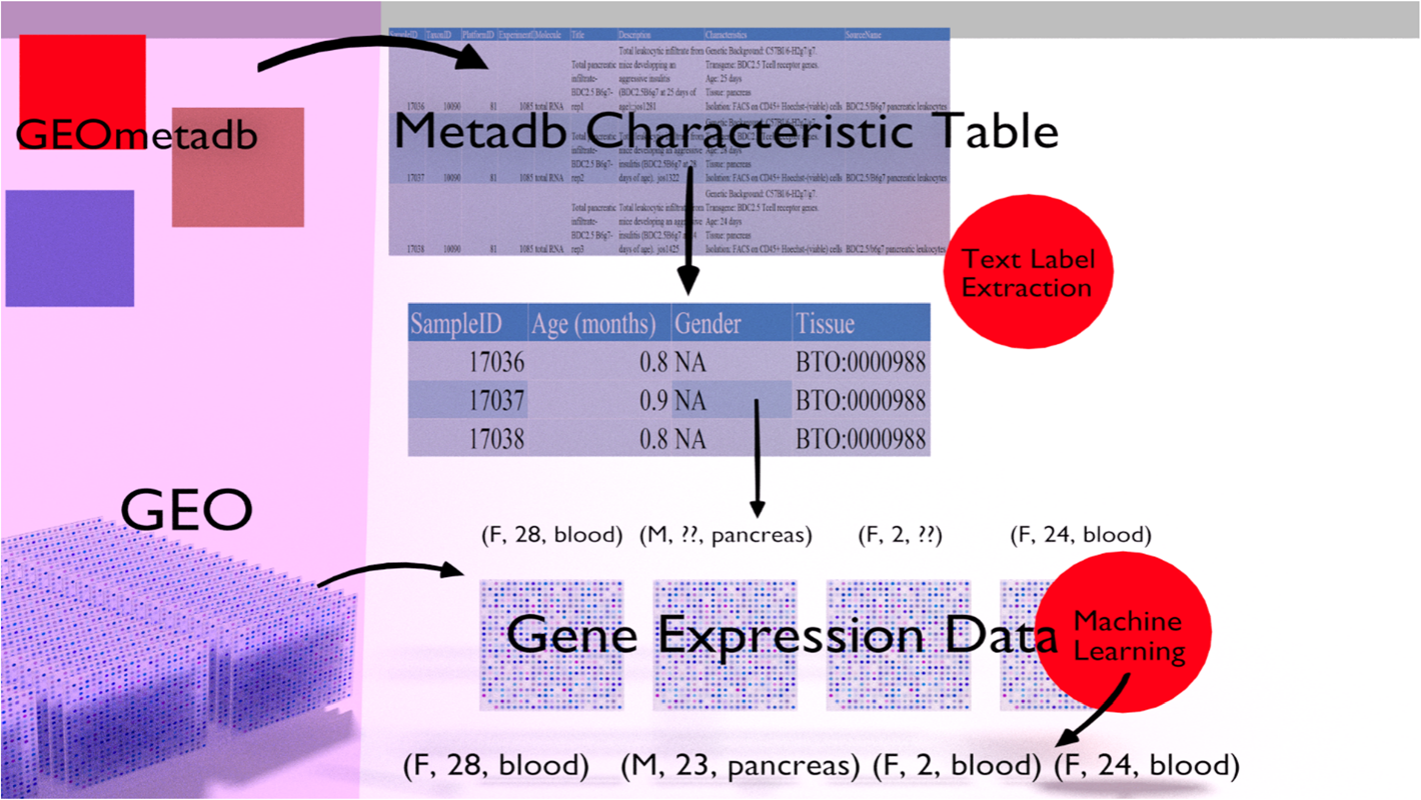
Graphical overview of the algorithmic process

A graphical overview of our algorithmic process is shown in Fig. 3.

### GEO expression data and metadata

Human gene expression data (159,370 samples from GPL570 and GPL96) were downloaded from GEO and values log transformed (if not already log transformed). Probes were collapsed to gene-level (Entrez Gene ID) by choosing the probe with the highest mean expression per gene, and normalized between arrays by quantile normalization. Imputation of missing values was done using k-nearest neighbors with k = 5. Metadata text for the downloaded GEO data was obtained from the GEOmetadb package [9] which contains several key fields with the label and experiment types of interest, such as “Title”, “Source Name” (usually referring to the tissue or cell line), “Organism”, “Description”, “Characteristics” (key value pairs denoting the attributes of the sample), “Molecule” (denoting whether the sample is DNA, RNA, poly-A RNA, etc.), and several other fields such as sample and platform ID. This database was queried using the SQLite command-line client for the fields “title”, “description”, and “characteristics chN”, where N is the microarray channel number (one-color arrays, which comprise most of GEO, will have one channel, whereas the older two-color arrays will have two). A typical “characteristics” field with its key-value pairs is provided in Table 1.

**Table 1:** Characteristics field example from a GEOmetaDB sample (GSM17122)

Key	Value
*Genetic Background*	NOD
*Transgene*	BDC2.5 T cell receptor genes
*Age*	25 days
*Tissue*	Pancreas
*Isolation*	FACS on CD45+ Hoechst- (viable) cells

In terms of problem difficulty, we hypothesized that gender would be the simplest label to extract using text-mining methods, and would therefore yield the highest performance, because of the limited number of possible values and limited number of ways it can be described. Similarly, in GEO metadata, age is generally clearly signaled by an “Age:” prefix, but there is additional difficulty because a variety of units can be used (months, years, etc.), sometimes it is implicit in the wording (e.g., “patient X (34, F, non-smoker)”), and occasionally units of age are misspelled. Tissue extraction should be the most difficult problem, because there are thousands of potential tissue types or cell lines, with a variety of synonyms.

### Heuristic extraction of labels from text

In order to extract labels from the unstructured metadata text, two broad approaches are used. For sex and age extraction, simple regular-expression based approaches are used, because these label types consist of a small vocabulary and the presentation of this type of data is relatively consistent within the metadata. For tissue extraction, a string-matching approach is used to map metadata to ontology term names and synonyms. For all problem types, the parsed “Characteristics” fields are searched first, because they contain attributes the experimenter has explicitly labeled as such, and if a match is found for that label-sample combination in the “Characteristics”, the search is terminated. Otherwise, it continues to the other fields in the order “Description”, “Source Name”, “Title”, and finally, all other fields.

### Sex labels from text

Heuristic extraction of gender was performed using regular expressions searching for patterns such as “gender: male”, “sex: M”, or “sex: 1” for numerical, abbreviations, or complete text encodings.

### Age labels from text

Similarly, extraction of age was also performed using regular expressions; however, in this case, the regular expression also attempts to extract the units in text such as “age: 29 y”, “age (mo): 520”, etc. Where an age number was extracted, but not a unit, a default unit was assigned depending on the species. For humans, this is years, but the software can also extract labels for nonhuman species, although the current paper does not address this topic. In the case of rodents, for example, the default is months.

### Tissue / ontology labels from text

**Fig. 4:**
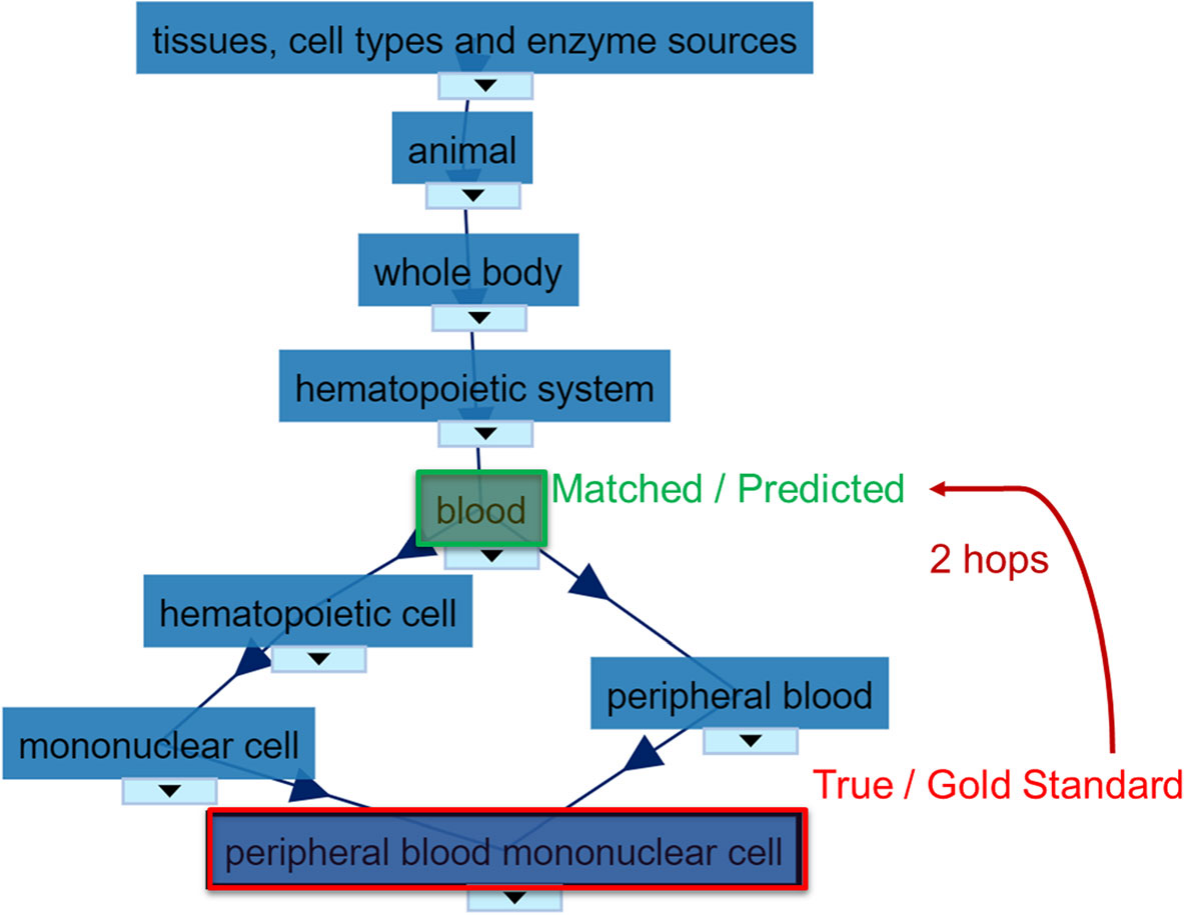
Illustration of matched terms in BRENDA ontology. Created with the visualization package in bioportal.bioontology.org

To extract tissue types, the metadata was searched for term names or synonyms from the BRENDA Tissue Ontology (BTO) [10] using the Aho-Corasick multiple string search algorithm [11]. In the case of multiple matches, the node which is shallowest, or most general, in the ontology was selected; while this may lead to some uninformative matches, we chose to focus on high accuracy annotations rather than potentially erroneous matches from a less conservative approach. Preliminary experiments showed that this approach yielded better performance than choosing the most specific node (data not shown). This is likely because the BRENDA Tissue Ontology contains many short-term synonyms such as “bud”, “cap”, and so forth which lead to false positives. Figure 4 shows an example of the BRENDA ontology structure.

### Label prediction from gene expression data

In addition to the extraction of labels from text, we perform inference of labels from gene expression. Because the problem types are different, we use different classifier/regression algorithms for each label type. The input data to the classifier is gene expression data normalized as described above, and the most informative 100 genes are selected to reduce dimensionality. To predict gender and tissue type, we used one-vs-rest (OVR) logistic regression.

The choice between multi-class (one-to-one mapping) vs multi-label (one-to-many mapping) is important, as some samples may be comprised of multiple tissues, and therefore will be more suited to a multi-label approach. However, the benefit of using the BRENDA Tissue Ontology is that it contains nodes for tissue/cell types, such as blood, that are actually composed of multiple cell types, yet one node may imply many labels that are associated with the parent node within the ontology structure. This helps reduce the problem to a multi-class problem, wherein the single parent node may be used to retrieve any tissue belonging to this tree structure. Therefore, we utilize this ontological structure to reduce the problem to that of a multiclass problem and consider the tissue prediction in a multiclass context.

Expression matrices were pre-processed using two feature selection methods, first using scitkitlearn’s *VarianceThreshold()* function to exclude genes with the low expression variance, then *f_classif()* was utilized to pick the top 100 F-scores among the remaining sites for downstream usage. Lastly, *LogisticRegression()* was used to predict the probability a sample belonged to any given feature (such as tissue).

### System design

To extract metadata labels from GEO, our system uses both the textual metadata provided by the investigator with each sample, and the sample’s expression data itself. It first applies pattern-matching algorithms to the investigator-supplied textual metadata to attempt to extract each label type (e.g., age, gender, and tissue type). If this fails, either because the pattern-matching was insufficiently robust or because the investigator did not provide the label, the system uses a machine-learning classifier, trained on manually curated labels or labels extracted from pattern-matching, to predict the label from that particular sample’s gene expression vector itself, similar to ontology-mapping OVR SVM approaches used in previous works [4]. At the outset, we hypothesized that using pattern-matching based approaches on the textual metadata would result in greater extraction precision than machine learning approaches, at the expense of recall. This is a typical tradeoff for heuristic approaches [12]. By combining the two approaches, we intended to achieve a better overall balance between precision and recall.

### Gold standard

We manually labeled 38,188 samples from GEO from human gene expression experiments with gender, age, and tissue (using the BRENDA Tissue Ontology [10]). This data and the characteristics of the data are summarized in Table 2.

**Table 2:** Gold standard - Summary of manually annotated samples

	Gender	Age	Tissue
*Samples Annotated*	17,065	9696	38,099
*% Samples Containing Label*	44.7%	25.4%	99.8%
*Most frequent label (mean for age)*	Male (52.7%)	46.7 (SD 21.6) years	Lung (6.8%)

## Results

The evaluation of performance for each method was determined using manually curated gold standard labels, as described in the methods. To train the machine-learning classifiers, we used the labels extracted from the heuristic approach which were not part of the manually curated set, and tested the results against the manually curated labels, using 10-fold cross-validation. A summary of the labels extracted with the heuristic approach is given in Table 3.

**Table 3:** Training data - summary of the labels extracted using the heuristic (regular expression based) approach, which were supplied to the machine learning algorithm as training data

	Gender	Age	Tissue
*Samples Annotated*	441,311	299,878	861,703
*% Samples Containing Label*	51.2%	34.8%	100.0%
*Most frequent label (mean for age)*	Female (50.3%)	51.0 years	Blood (12.5%)

### Label extraction for tissue, age and gender

**Fig. 5:**
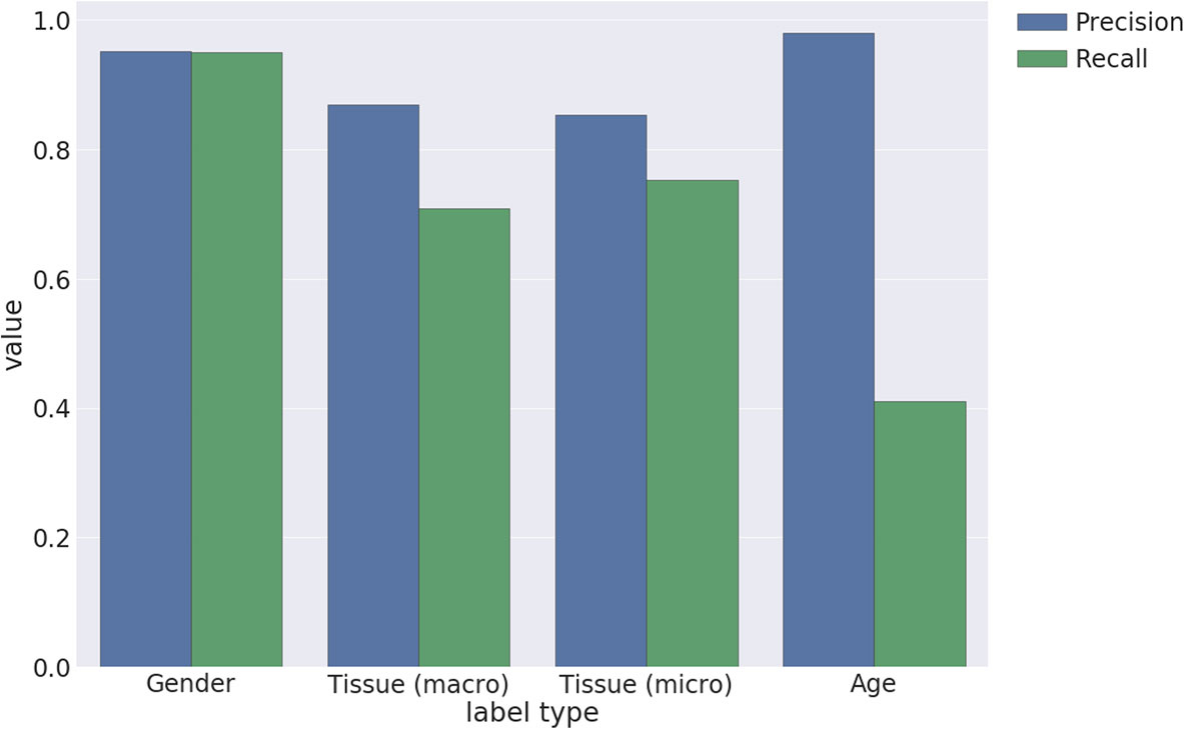
Precision-recall metrics for extracting age, gender, and tissue labels from text. Macro-average and micro-average metrics are shown for tissue label prediction, using the top 25 most frequently occurring labels in the gold standard. For Age, if an age value was extracted, it was considered as a true positive if the extracted value was within 1 month of the gold standard value, otherwise a false positive

The results demonstrating the performance of each label category are summarized in Fig. 5. The gender label category achieved a precision/recall of 0.94/0.98 for males and 0.95/0.90 for females using the heuristic method for extraction. Age was extracted with a mean absolute deviation (MAD) of 1.01 years, a mean squared error (MSE) of 225.6 years. In general, errors for age were rare (98% of samples with extracted values had results within 1 month of the correct value), but generally when errors occurred, they were large in magnitude and resulted from a failure in time unit conversion. Many of these erroneous extractions can be excluded in practice by bounding ages within realistic human age intervals. Recall for age was considerably lower than that for gender.

**Fig. 6:**
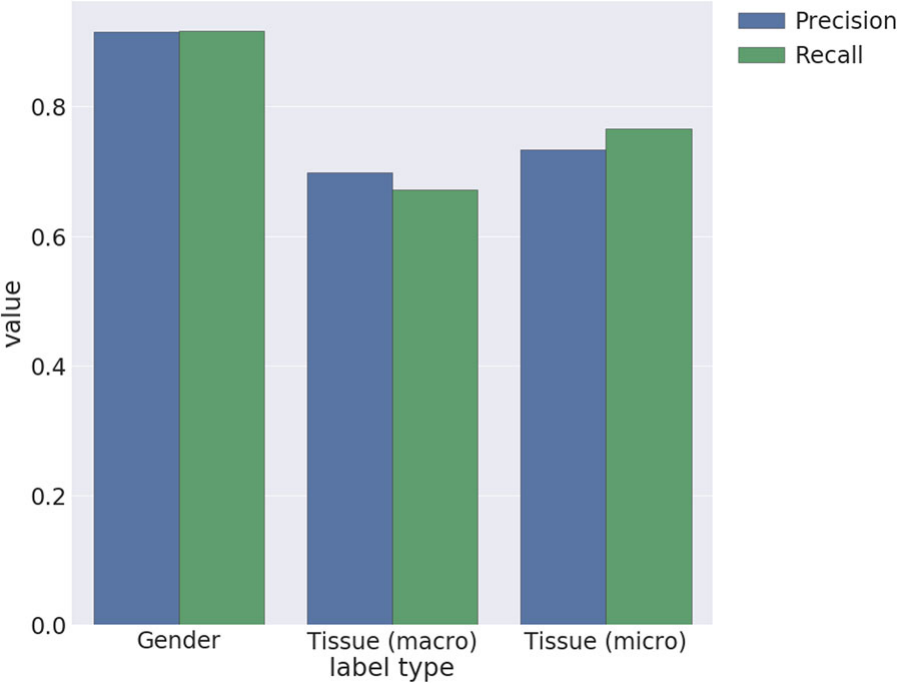
Precision-recall metrics for the gender, and tissue label types for the expression-based machine learning predictions. Age was not predicted with gene expression. Macro-average and micro-average metrics are shown for tissue label prediction using the top 25 most frequently occurring labels in the gold standard

**Fig. 7:**
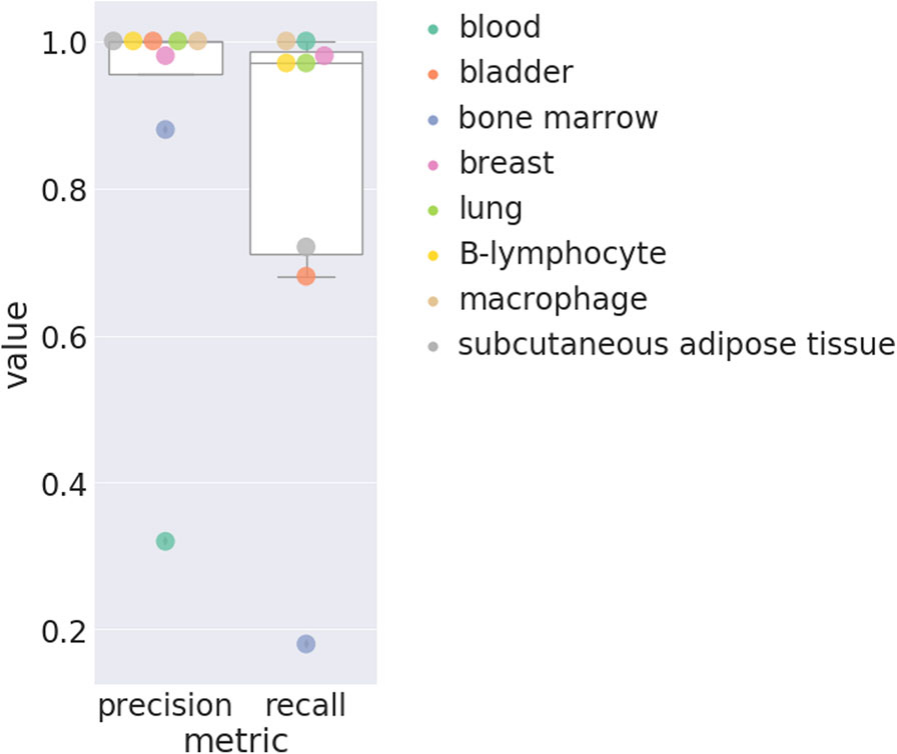
Per-tissue precision and recall for the tissue prediction results

**Fig. 8:**
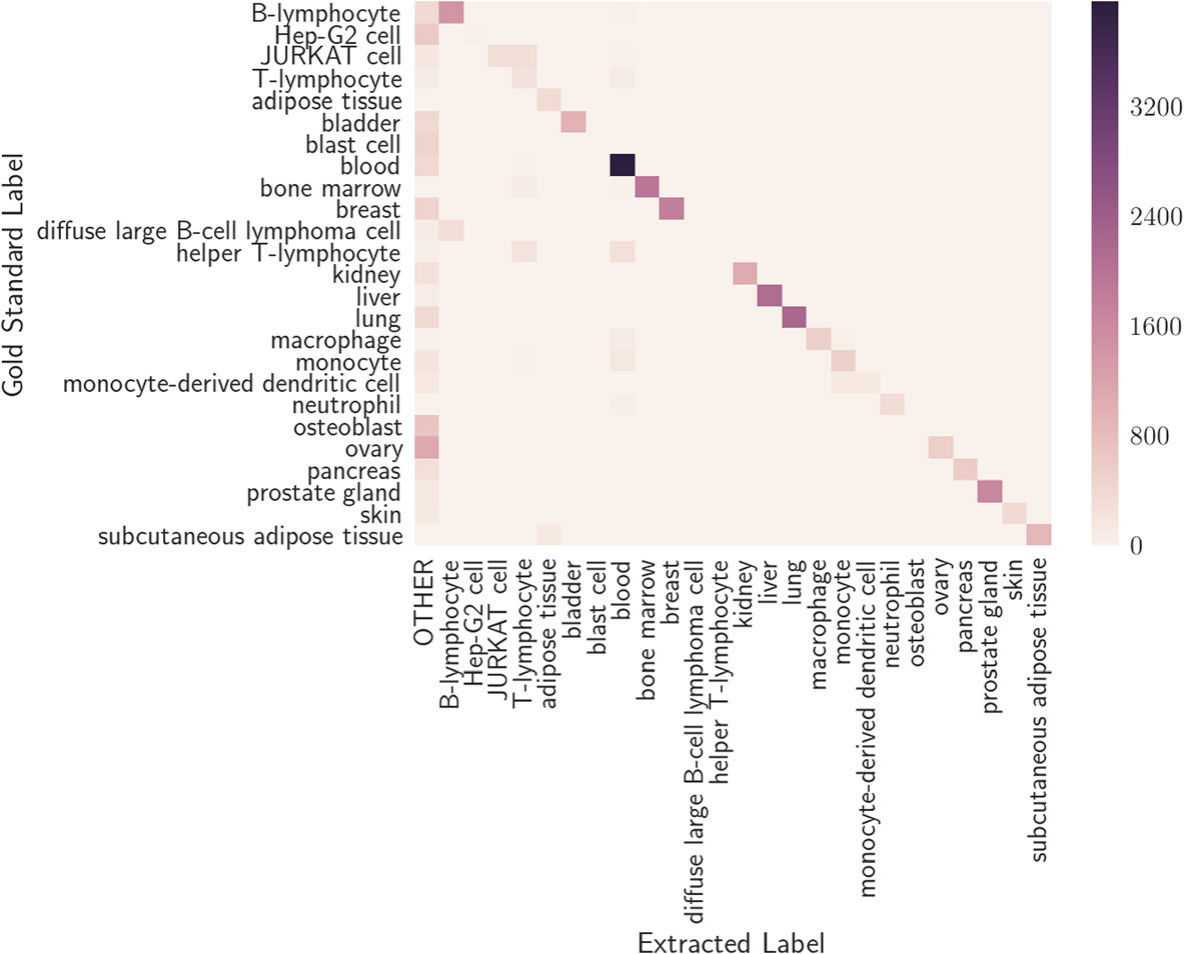
Confusion matrix of top 25 most common tissue labels in the gold standard data

Tissue label extraction performance in Fig. 6 shows the precision for both *micro* and *macro* averaged tissue to be similar, while the recall diverges. Micro-average precision and recall over the 108 tissue types which had at least 10 samples was 0.77 and 0.54, whereas within the top 25 most frequent gold-standard tissues, it was 0.85 and 0.75. Figure 7 shows the precision and recall for a selection of common individual tissues and a confusion matrix for the predictions on the top 25 most common tissue labels within the BRENDA Tissue Ontology is shown in Fig. 8.

### Label prediction from GEO expression

The ML approach was able to predict gender from gene expression data with a high macro precision (0.915) and recall (0.917). Tissue was not as accurate, but during cross-validation on the top 25 most frequently occurring tissues, we achieved a macro precision of 0.70 and macro recall of 0.67. Micro-precision was 0.73 and micro-recall was 0.77. The macro AUC for ML classification was 0.83, contrasted with a baseline 0.49 for a naive “dummy classifier”, which makes predictions based purely on the distribution of training class labels (without using expression data).

## Discussion

### Label extraction for tissue, age and gender

The extraction of labels from the textual metadata was performed using pattern matching, which is appropriate for tuning performance metrics towards high precision at the expense of recall. This choice allows for more certainty in the results, which are used as training data provided to the GEO expression machine learning algorithm. While the precision and recall within gender and tissue are within ~20%, the recall for age is noticeably lower than gender. We attribute this to a greater variation in the patterns used to describe age, and to the fact that the variable placement of the age unit often interferes with heuristic extraction.

**Fig. 9:**
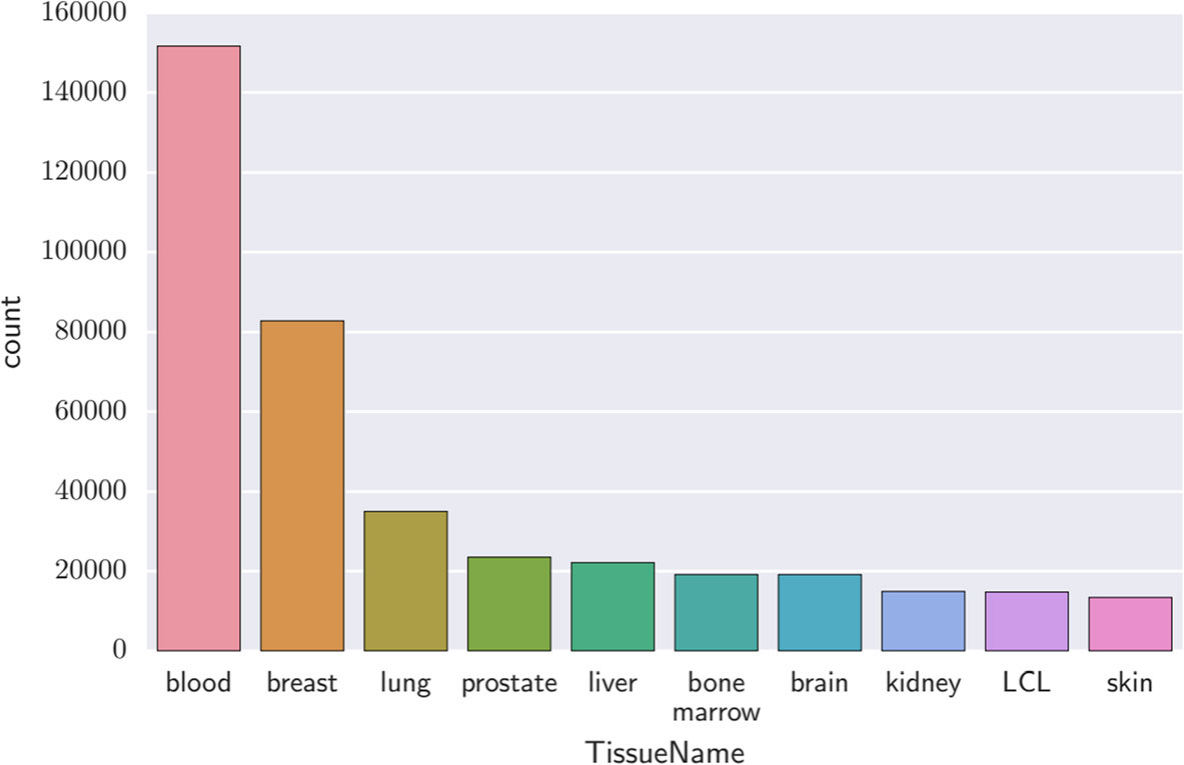
BRENDA ontology categories for the top 10 most common tissues in GEO

Tissue label extraction performance is evaluated in terms of *micro-* and *macro-* precision and recall, whereby micro-averaging will be the overall precision/recall across all predictions, not taking into account label imbalance (Fig. 9 shows how tissue type labels are imbalanced), and macro-averaging is the average of precision/recall across labels.

The imbalance of tissue type prevalence within GEO shown in Fig. 9 affects the micro-precision and micro-recall, as decreasing the number of tissues considered from the most common 108 tissues to the most common 25 tissues increases the precision and recall by 8% and 21%, respectively. This divergence suggests the method performs better on the more common tissue types. Fig. 7 displays the precision and recall for individual tissue types selected from some common tissues, which indicates the precision and recall tend towards higher values for samples which are more frequently occurring within the data, and therefore have more samples for which the algorithm may train.

**Fig. 10:**
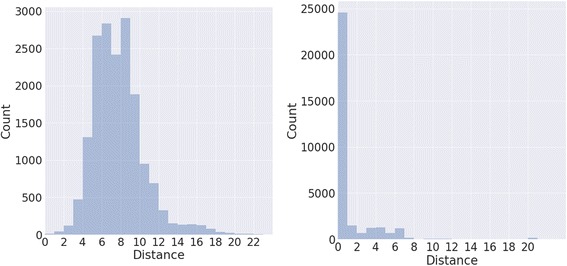
Ontology distance between extracted and gold standard tissue labels. The histogram on the left displays the distance distributions between randomly selected nodes and that of the gold standard, whereas on the right side is the distribution of shortest path lengths between the predicted label and the gold standard label using our approach

We also evaluate the distribution of distances from the predicted label node to that of the correct label node within the ontology. This provides a more complete view of the errors within the system, as a match between *hippocampal pyramidal layer* and *hippocampus* is “more correct” than *hippocampal pyramidal layer* matching to *hematopoietic system*. The distances measured between predicted tissue ontology nodes from GEO metadata and their gold standard label nodes is shown in Fig. 10, whereby a distance of zero indicates the predicted ontology category is the same as the gold standard ontology category. It can also be seen that the spread of distances is far reduced compared to randomly selected BTO IDs.

In order to get a more detailed understanding of the common tissue labels and the predictions made, we constructed a confusion matrix (Fig. 8) of the top 25 most common tissue labels within the BRENDA Tissue Ontology. Some of the complexity in accurate label identification can be seen in this figure. For example, there is a variety of ways an author can write “diffuse B-cell lymphoma”, and so the least variant term “B-cell” will tend to be identified as the best match. Synonyms also tend to be problematic, such as “ovarian” not being correctly mapped to “ovary”. This turns out to be due to the direct string matching algorithm used, which was chosen for speed (in order to process the large amount of data available in GEO) and because it allows for high precision at the expense of recall. It is assumed that, due to the large amount of data available, the low recall will still provide an ample number of samples from the entire GEO database, and therefore still allow for decent insights to be drawn from the data. Further improvement of false-negative rates will require either fuzzy string matching (which could increase false-positives) or manual expansion of synonyms for tissue types.

### Label prediction from GEO expression

We evaluated the results of the ML-based approach using 10-fold cross-validation stratified on experiment, using the data extracted from the text mining approach. That is, all samples from any individual experiment were either used for training or testing in any particular cross-validation fold. This is very important, as samples within any particular experiment tend to be highly similar to each other, relative to other samples in GEO, and thus cross-validation without this type of stratification will overestimate performance, as the classifier will be able to indirectly predict “labels” by in fact predicting membership in a particular experiment. In addition, this procedure enabled us to be more confident that a given classifier can generalize to unseen experiments. The following results were computed on the subset of samples from GPL96 which fit two criteria: a) annotated with the label type under query (gender or tissue), and b) expression data was available. Additionally, for tissue, evaluation was performed only on the top 10 most frequently occurring tissue types, as assessed during text-based label extraction. In total, cross-validation was performed on 10,129 samples for gender and 13,427 samples for tissue.

The choice to evaluate tissue label predictions for the most frequently occurring tissue types is due to lower micro-precision and micro-recall (0.73-0.77) received for these performance metrics. The lower relative cross-validation performance on tissue likely reflects several issues. First, a multiclass problem with more classes is an inherently more difficult classification task. Secondly, our feature selection approach of selecting the most informative 100 genes for downstream training and classification may be more applicable to extraction of gender, which is reflected largely by expression of X and Y chromosome genes, than the case of tissue, as tissue-specific or tissue-predictive genes are more heterogeneous and scattered throughout the genome, therefore 100 genes may not be sufficient for this task. Finally, errors during text-based label extraction will be propagated to some extent to the classification stage. This last issue highlights the importance of attaining maximum accuracy during the initial text extraction phase to maximize performance on the downstream ML classification stage, and we would expect precision improvements in the text extraction method to result in improved ML classification performance.

### Further applications

Although the current study is limited to a relatively constrained set of three types of labels, it lays the groundwork for a broader range of label type. For example, many other label types of interest, such as disease state, drug application, or diet, are specified in other ontologies, and our ontology-based approach could be applied with minimal modification to novel label types. Although we have not assessed performance on other label types, the code library for label extraction provides generic methods for extracting labels from any ontology in Open Biomedical Ontology (OBO) format.

Similarly, although the current study is limited in scope to expression data from humans, the text-based approaches in particular may be suitable for future extraction of labels from other species and data types, because the format of metadata is relatively independent of the underlying type of high-throughput data in GEO or the species. However, in the process of developing this tool, we did anecdotally notice minor differences between metadata between species, which would require additional work to make the tool fully applicable to model organisms such as mouse. For example, while human ages are usually expressed in terms of years, there is much more variety in terms of the time unit used to express mouse age: days, weeks, and months are all common, making the accurate detection and conversion of time unit difficult but necessary to properly expand our tool to model organisms. We also observed that the distribution of tissue type is somewhat different in humans compared to model organisms, in the sense that tissue samples derived from non-invasive procedures (such as blood samples from a blood draw) are relatively more common in humans compared to tissues, such as brain, that can only be obtained from invasive procedures. These observations imply that evaluation metrics and extraction methods would likely require additional tuning to apply fully to model organisms.

Previous work by other authors, e.g. [4], has leveraged the directed acyclic graph (DAG) structure of ontologies to share information from tissues or other ontological terms which are conceptually or structurally related, but not identical. For the purposes of simplicity, we did not pursue such an approach in this work, but we expect that such an extension would prove especially valuable in the case of label prediction from gene expression, as it seems reasonable to expect that related tissues, disease states, etc., would be related in terms of gene expression as well. Thus, for example, if an expression-trained classifier assessed a high probability that a sample should be assigned the label “whole blood”, the likelihood that the sample is comprised of PBMCs would likely be consequently higher; i.e., nodes in an ontology structure would be expected to be somewhat conditionally dependent.

## Conclusion

We have presented a tool for the extraction of gender, tissue, and age labels for GEO data from the associated metadata, as well as a label prediction tool to probabilistically assign missing labels based on gene expression data. Broadly, we found that relatively simple heuristic text-extraction approaches based on regular expression and string matching can identify labels, especially those consisting of small vocabularies, such as age and gender, with high precision and moderate recall. The labels from heuristic extraction can then be used to provide larger training sets to train ML models on expression data to expand the range of samples that can be categorized, in case heuristic extraction fails, or the sample metadata does not contain the required information. Thus, by combining the two approaches, we retrieve accurate labels without sacrificing too many samples (maintaining precision and improving recall), greatly enhancing the ease by which large scale analysis can be performed using GEO data spanning across many studies. Another advantage of this two-pronged approach is that ML models can be trained on newly released transcriptional profiling platforms without the need for manual annotation of labels for each individual platform.

## Abbreviations

ALE: Automated Label Extraction Software
AUC: Area under curve (for the ROC, or receiver operating characteristic)
BTO: BRENDA Tissue Ontology (BRENDA being originally an acronym for Braunschwieg Enzyme Database)
DAG: Directed acyclic graph
GEO: Gene Expression Omnibus
OVR: One versus rest (type of support vector machine)
SRA: Sequence Read Archive
SVM: Support vector machine
URSA: Unveiling RNA Sample Annotation
